# Using expert knowledge and peer review to create a reproducible process for the NAHRS Nursing Essential Resources List (NNERL)

**DOI:** 10.5195/jmla.2025.1964

**Published:** 2025-01-14

**Authors:** Rebecca Raszewski, Lorraine Porcello, Alissa V. Fial, Carolyn C. Dennison, Rachel Keiko Stark, Karen S. Alcorn, Sarah Wade

**Affiliations:** 1 raszewr1@uic.edu, University of Illinois Chicago, Library of the Health Sciences Chicago, Chicago, IL; 2 Lorraine_Porcello@URMC.Rochester.edu, University of Rochester, Rochester, NY; 3 alissa.fial@marquette.edu, Marquette University, Milwaukee, WI; 4 cdenniso@hawaii.edu, University of Hawaii at Manoa, Honolulu, HI; 5 stark@csus.edu, Sacramento State University, Sacramento, CA Karen S. Alcorn, MLS; 6 karen.alcorn@mcphs.edu, Massachusetts College of Pharmacy and Health Sciences, Worcester, MA; 7 swade@campbell.edu, Campbell University School of Osteopathic Medicine, Lillington, NC

**Keywords:** Library Collection Development, Libraries, Nursing, Case Reports, Nursing and Allied Health Resources and Services (NAHRS)

## Abstract

**Background:**

Librarians have relied on resource lists for developing nursing collections, but these lists are usually in static or subscription-based formats. An example of this is the 26th edition of the Essential Nursing Resources last published in 2012. The Nursing and Allied Health Resources and Services (NAHRS) Caucus Nursing Essential Resources List (NNERL) Task Force has been working on a new list since Fall 2020. The goal of the Task Force is to create a nursing resource list that represents current materials and formats, uses a selection process that is transparent and reproducible, and will be available to a broad audience.

**Case Presentation:**

Working from the Essential Nursing Resources 26th edition, the NNERL Task Force updated the purpose statement then began reviewing the resources on the list. Two working groups were formed: 1) an evaluation rubric working group developed a tool to evaluate the resources and 2) a tagging work group developed guidelines for creating metadata and “tags.” Volunteers were recruited from the NAHRS Caucus to tag the resources. Lastly, the Task Force finalized the list of resources in the NNERL then cleaned and reconciled the data.

**Conclusions:**

The final version of the NNERL will be published in Airtable, a cloud-based project management product, that will include metadata for every item on the list. The NNERL will be copyrighted to the NAHRS NNERL Task Force and made available through the Open Science Framework (OSF) under an Attribution-NonCommercial-NoDerivatives 4.0 International Creative Commons License.

## BACKGROUND

Resource lists of books, journals, or websites for the nursing profession have existed for decades. However, until now, these lists were either one-time publications, no longer updated, or required a paid subscription [1–6]. Librarians are often involved in creating these lists, using their professional knowledge to ensure the recommended materials are current and of high quality. Recommended resource lists help healthcare students and professionals find reliable and trusted resources for their studies, practice, or scholarship [7, 8]. While these lists have been helpful,, there is no evidence in the literature they are created in a reproducible and peer reviewed manner.

This case report describes the processes developed by a task force from the Nursing and Allied Health Resources and Service (NAHRS) Caucus of the Medical Library Association (MLA) to create the NAHRS Nursing Essential Resource List, hereafter called the NNERL. The processes included developing a rubric-based evaluation of nursing information resources, a vocabulary of metadata “tags” to describe each resource, and a structured review procedure. Through these improvements, the NNERL is a new resource that is accessible to all, regardless of professional membership. This case report also provides a model for similar groups within MLA to create or sustain collection development resources in other health sciences disciplines. By peer reviewing the materials included in the list and creating a reproducible methodology for the creation and maintenance of this list, the NNERL Task Force seeks to create a high-quality resource that follows current standards for open science, while being easier to update and maintain than traditional lists.

### The Essential Nursing Resources List

In 1966, the Interagency Council on Information Resources in Nursing (ICIRN) first published the Essential Nursing Resources List (ENRL) [9]. Expert information professionals with experience and responsibilities in nursing librarianship created the ENRL as a curated list of core resources for nursing libraries to utilize for collection development, current awareness, professional education, and career advancement. Schnall and Fowler published the 26th and final edition of the list in 2012 [10]. The ICIRN disbanded in 2017.

In October 2020, the NNERL Task Force leader recruited a team of volunteers from NAHRS to continue the ICIRN's work. Rather than follow the previous format and workflow, the Task Force aimed to reimagine the list as a living document and develop consistent methods for future updates. The Task Force leader charged two working groups to accomplish these goals. One group of four members focused on creating a rubric for evaluating resources, while the other group of six members created metadata “tags” with associated definitions to describe the NNERL resources. Completing their respective charges, each working group created supportive documentation and reproducible methodology for evaluating, indexing, and adding or removing resources from the NNERL.

## CASE PRESENTATION

### Updating Purpose Statement

The 2012 edition of the ENRL included 396 items available through print, electronic, and mobile formats as a static list. It was “presented as a resource for locating nursing information and for collection development… to support nursing practice, education, administration, and research activities. The list was compiled to point to pathways for exploration, rather than be an endpoint, and to expand to other formats beyond traditional references” [10]. Blogs, forums, and discussion lists were added to this version. Due to the issues with the permanence of blogs, forums, and discussion lists, as well as evolving professional standards, these materials were removed from this version of the NNERL.

To reflect the extensive changes made to the 2012 ENRL and to acknowledge the role of NAHRS in taking over the list, the 2024 NNERL is considered a new version. However, the purpose statement remained the same, except for adding language that the NNERL is for information professionals, and items “on this list represent high quality, evidence-based, and/or peer-reviewed resources appropriate for use in scholarly work, clinical practice, and research” [11]. The NNERL Task Force added an Attribution-NonCommercial-NoDerivatives 4.0 International Copyright designation (CC BY-NC-ND 4.0), enabling list users to copy or redistribute the NNERL and its associated materials [12].

### Reviewing and Updating Resources

To begin, a Task Force member assigned each resource a numerical identifier and then moved all extant resources to a master spreadsheet. Next, each NNERL Task Force member received a subset of resources divided into blocks of roughly 25, with instructions provided for the initial review. Upon completion, each reviewer received a new set of resources until each resource was independently reviewed by at least two Task Force members. The instructions for the initial review included a color-coding system to indicate that a resource should be retained (green), removed (red), or required further discussion with the Task Force (yellow). During virtual monthly meetings, members discussed all resources that did not receive matching recommendations from the two reviewers, until achieving consensus.

In general, the Task Force's review of existing websites on the list resulted in retaining only the main website and removing the subsidiary sites. For example, the 2012 ENRL included the Centers for Disease Prevention and Control (CDC) homepage and links to subsidiary sites within the CDC.gov domain (e.g., CDC Wonder). Since website domain information deteriorates faster over time than information in other formats, the reduction of website subsidiaries aimed to minimize the impact of URL changes, which were assumed less likely for prominent organizations such as the CDC or WHO. For websites already on the list, only rarely and by consensus were subsidiary web resources retained.

### Rubric Creation

To ensure the reproducibility of the peer review process, the rubric working group created a document with 9 questions that addressed current needs and expectations for inclusion in the NNERL when applied to each resource. This rubric would be used to evaluate the materials for inclusion to reduce the likelihood the Task Force would inject bias during the evaluation process of the included resources. The Task Force recommended using literacy standards and the NNERL's updated purpose statement as a starting point for the working group, both of which informed the rubric's creation. The above-listed standards included resources on evaluating information and internet resources [13–15]. The NNERL's updated purpose statement guided the resource evaluation criteria. Each rubric characteristic included scope notes (i.e. brief definitions and/or examples for use), creating a shared understanding of terms. The rubric designated the following characteristics to evaluate each resource:

- Authors or creators of listed resource
- Transparency of methods for resource creation
- Expertise of authors or creators
- Date of creation and/or last update
- Frequency of updates
- Funding disclosure
- Conflict of interest disclosure
- Inclusion of historically underrepresented groups
- Relevance of the resource to health information professionals or a health sciences library collection

The rubric and the scope notes are available in Appendix A.

The NNERL Task Force used a small subset of NNERL resources to test the usability of the evaluation rubric. Four testers were recruited from within the Task Force for the first round of revisions, with three testers recruited from the larger NNERL Task Force for the second round of testing. To test the rubric, a small random selection of resources was assigned to testers, with each item being reviewed using the rubric twice, each time by a different tester. After the two reviewers' results were compared to ensure the rubric's usability, comments were solicited from all the testers. The rubric, once tested and completed, was then used by the NNERL Task Force to review each resource within the NNERL list. Each item in the NNERL list was reviewed by at least two different reviewers, and the results of those reviews were then used as part of the discussion for inclusion for each resource. Scores alone were not used to automatically accept or reject a resource. However, the scores collected for each item in the NNERL list will remain unpublished because they are not validated and only meaningful to the Task Force.

### Tagging and Scope Note Creation

A second NNERL working group focused on creating “tags” for the items included on the NNERL list. The NNERL Task Force used the term “tag” to refer to the metadata assigned to individual resources. “Tag” was chosen as the descriptive term that the working group used due to lack of better options, there is no connection to the use of “tag” in this document with the kind of tagging created in social media, hence why “tag” is in quotations. The goal of creating “tags” as a form of metadata was to make items on the list searchable. While the ultimate goal of the NNERL Task Force is to make the list available online, which would require metadata creation, having “tags” available for each item in the list in its current form allows users to use a find option for materials based on their “tags.” The working group developed a list of “tags” and a set of instructions to guide the process of assigning them. Then the “tags” were given to NNERL Task Force members to test by applying them to a random selection of resources from the NNERL list. Each item was reviewed by two volunteers, with feedback solicited from testers and modifications made based on feedback. The final list of “tags” included those from the 2012 ENRL, and newly created “tags” to reflect current trends, formats, and use. Next, the working group organized the “tags” into four categories: format, cost, areas of interest, and archival. During the review, some format tags from the ENRL were removed for being too specific (e.g., dictionaries); other format “tags” were added to assist librarians with their collection development and management responsibilities. For example, cost “tags” in the ENRL were previously limited to “Fee Required.” The “tag,” “Free,” was added along with a new “tag,” “Freemium,” which aimed to identify resources that offer a “Free” version as well as a “Fee” version that includes more robust features and content for an additional cost. Areas of Interest “tags” (e.g., “Evidence-Based Nursing,” “Informatics”) from the previous edition were reviewed by the entire NNERL Task Force, with “tags” added and removed to reflect changes in the creation and dissemination of information. The Archival category of “tags” identifies resources that are no longer updated but have historical importance or significance for librarians (e.g., “multivolume sets,” “bibliographies”). Table One lists the major descriptive “tag” categories and their definitions. The full list of “tags” and their scope notes are available in Appendix B.

**Table 1:** Descriptive “Tags”

Category	Definition
**Format**	A version of the item in which it is available (e.g., book, database, journals, serials, web-based resources). A Format “tag” is applied to every item.
**Cost-Related to the Item**	Use for item related costs: free, subscription-based, or freemium. Freemium is used for a free version with robust features and content for an additional fee. The Cost related to the item “tag” is applied to every item.
**Areas of Interest/Topics**	Descriptive terms are used to identify the subject content of each item. These “tags” are developed from MeSH (Medical Subject Headings) definitions and CINAHL (Cumulative Index of Nursing and Allied Health Literature) headings. Up to five “tags” are applied to every item.
**Archival**	Special “tag” only applied to items that are no longer updated.

A two-member team of the tagging working group created scope notes for each Area of Interest and Format “tag.” Another two-member team of the tagging working group reviewed the scope notes, and the two teams discussed disagreements in wording until reaching a consensus. Each team met virtually, with the two teams meeting together three times to finalize the scope notes. Once the tagging working group completed the scope notes for the “tags,” they developed instructions for assigning them. The guideline included in-depth information about each category and detailed scope notes for each Area of Interest “tag.” The Task Force leader solicited volunteers from the NAHRS Caucus to test the guidelines for face validity [16] and inter-rater reliability. Each resource had two testers. The “tags” and the guidelines were adjusted based on the testers' feedback. For example, the testers suggested adding more “tags,” particularly for areas of interest that were not part of the previous list (e.g., Multidisciplinary).

### Final Review of Resources and Data

Spreadsheets were used in the development stage of the NNERL to ensure all members had access to the materials used by the group; spreadsheets were accessible to all members regardless of institutional limitations; however, the Task Force did not want the NNERL to remain in a spreadsheet format.

After the resources were scored and tagged, each resource's metadata was consolidated into one spreadsheet to clean and reconcile the data. NNERL Task Force members collaborated on updating the metadata and writing descriptions for each resource. They marked resources that were not deemed as essential to nursing for discussion by the entire Task Force during their monthly meetings. Several resources were removed during this process (e.g. Drugs@FDA, National Science Foundation), and a few more were added based on feedback obtained during reviewing the resources' “tags” (e.g. Nursing: Scope and Standards of Practice and Scopus). Next, the team selected which information would remain in the public version, and which would be archived such as raw rubric scores for resources. Finally, they standardized the data in each column to ensure consistency between entries. Once the spreadsheet was clean and usable, the “tags” were incorporated into the spreadsheet and reviewed.

### Designing End Product

The Task Force's work on the NNERL incorporated best practices in collection development, assessment, and data management. Previous sections of this case report demonstrated the attention paid to choosing and evaluating information resources essential to supporting the work of nurses and nursing librarians. Of equal importance are the tenets of data management. Kipps & Jones point out that librarians routinely support researchers in designing research data management plans; however, “there are other data that are crucial to the library workplace. These include usage data, quantitative data … financial data, circulation data, and more” [17]. The NNERL project is a rich source of factual and descriptive metadata.

As such, NNERL Task Force members with experience in database creation and management explored options for transforming the cleaned list from a static spreadsheet to a searchable database. They focused on solutions that would address such requirements as an accessible and searchable interface, adequate data storage capacity, scalability to accommodate future growth of the list, robust tutorials, or support information, at little to no cost. Unsurprisingly, resource-intense tools such as a Structured Query Language (SQL) server to access data needed to meet additional requirements, so a cloud-based solution became the focus of the investigation. Cloud-based project management options that are available today combine data storage with functionality essential for teams whose members collaborate remotely. One such out-of-the-box solution is Airtable, which combines the features of spreadsheets with database functionality but does not require extensive prior knowledge to use efficiently [17]. More importantly, Airtable uses a freemium pricing model wherein the cost is not incurred until the database grows beyond 1,000 records. While it is notable that certain interface customizations are not available on the no-cost plan, Airtable is the current solution for the NNERL Task Force because of its data visualization and project management features.

Although an Airtable database, known as a “base” [18], can consist of many tables, the NNERL base currently has only one table. Future growth areas include adding tables for data related to NNERL. For example, when items are eventually removed from the public-facing table, they can be archived in a separate table accessible by designated individual(s) within the NAHRS Caucus.

Two team members initiated the transition from an Excel spreadsheet to Airtable base. They entered a subset of 60 records from the master spreadsheet into a database to learn how to use Airtable and test the usability of the spreadsheet data. The spreadsheet's columns are fields in the database, and each resource on the list is a record. The efforts invested in cleaning the data while still in spreadsheet format yields benefits because there is consistency between records for each field in the table. Plans to create visualizations of the NNERL records have been initiated beyond what is available in the grid-only view of a spreadsheet. For example, Airtable provides a “kanban view” that groups items into stacks of information cards based on a specified field [19]. Thus, grouping records by fields, such as format or cost, takes minutes. Customizing the visualization of NNERL records involves grouping, sorting, and color-coding records and fields but can also include hiding or filtering data [20]. For example, the rubric scores for each resource are not independently validated; as such, they are meaningful only to the Task Force. Filtering out these fields from the public view does not delete the data; but prioritizes the information needed for collection development.

A link to the NNERL through Airtable will be posted on the Open Science Framework (OSF) repository. A static version of the NNERL will also be provided on OSF for those working in settings with strict firewalls and network security.

## DISCUSSION

The NNERL is an updated list of peer-reviewed and high-quality resources that can be used to develop solid and usable collections of nursing resources. By making the list freely available, information professionals, nurses, and those who work at nursing schools worldwide can rely on the NNERL to find appropriate resources. This case report represents the first step in the dissemination process, to be followed by communication to multiple health science librarian newsletters and listservs. Raising awareness of the NNERL with health sciences librarians in academic and hospital settings enables them to share it with their nursing faculty, staff nurses, and clinicians. Furthermore, targeted submissions to newsletters of nursing organizations such as the American Nursing Association and other nursing specialty organizations, will broaden the reach of the list.

### Limitations

As with any task force, relying on unpaid volunteers can be challenging. Some librarians initially interested in working on the NNERL left the Task Force due to other time commitments. The final group of Task Force members were in different time zones across the United States (including Hawaii), so they were limited to when they could meet. Despite the challenges inherent in online collaboration across six US time zones, the Task Force assigned work to individuals or two-person teams who would provide progress reports during monthly meetings on Zoom. Eventually, the workflow evolved to include separate resource review meetings; although not required for the whole group, anyone working on assigned tasks could use these meetings to ask questions, resolve issues with specific resources, and determine the next steps. These meetings always generated rich discussions on collection development for nursing collections and which resources were considered essential.

The COVID-19 pandemic was a significant barrier for the work of the Task Force. Members tried to complete tasks alongside their primary job responsibilities, while adjusting to working remotely. Scoring print resources was particularly challenging because of multiple COVID-19 facility closures, which contributed to further delays in reviewing resources. Finding an online collaborative tool for working on documents together is an area for improvement. Although the Task Force selected Microsoft Teams, most members working remotely were using personal devices that did not allow access to Teams. Thus, team members received individual spreadsheets by email to update metadata or provide consolidated rubric scores. Merging multiple spreadsheets significantly complicated the data-cleaning process.

### Future Plans/Moving Forward

After constructing the public interface, the next step is to create data entry forms using configuration tools available in Airtable. Once these tasks are completed, the NNERL is ready to succeed as the newest version of this valuable collection development tool. The commitment of the Task Force to reproducibility and transparency allows a documented framework for future groups to update the NNERL, which will maintain its quality and usability.

The NNERL Task Force developed a Leadership Position Description for a NAHRS Caucus member to serve as the overseer of the list. The expectation is the individual will serve in the role for two years. During that time, the individual will be a contact person for the Airtable platform, and quarterly elicit feedback on items currently in the list and additional items to be added. They will also seek volunteers to coordinate these efforts; utilizing the process developed by the Task Force. In addition, the Task Force will furnish NAHRS Caucus leadership with documentation for maintaining and growing the NNERL in years to come.

## Data Availability

The rubric, “tags,” a link to the NNERL in Airtable, and a static version of the NNERL will be posted on the OSF repository: https://osf.io/urjyz/?view_only=d3f4e0f57b864ee4838ae66b95eac858.

## SUPPLEMENTAL FILES

**Appendix A:** Rubric

**Appendix B:** “Tags” with scope notes

**Appendix C:** Additional Acknowledgements
