# Supplementary material for: Using expert knowledge and peer review to create a reproducible process for the NAHRS Nursing Essential Resources List (NNERL)
Source: J Med Libr Assoc. 2025 Jan 14;113(1):78–84. doi: 10.5195/jmla.2025.1964 (PMC11835036; doi:10.5195/jmla.2025.1964)
Supplement: Supplementary file 1 — Appendix A: Rubric [file jmla-113-1-78-s01.docx]

# Appendix One: Rubric

## Q1. Is the author(s)/ creator(s) of material(s) provided in/on resource?

(1 pts = anything provided i.e. organization or individual authors; 0 pts = for nothing)

## Q2. Are the methods for how the information was gathered and/or used transparent?

(0 pts = no information; 1 pts = limited or tangential information; 2 pts = descriptive and more complete information)

### Scope Note for Q2.

- Databases:
  - 2 pts for full disclosure of how the search results are ranked and information on how the materials included in the database are chosen for inclusion;
  - 1 pts for disclosure of how search results are ranked OR for information on how materials are included in the database for inclusion;
  - 0 pts for no information for how search results are ranked and/or information on how materials are included.
- Electronic Resources (online websites, web portals, curated resource lists, Research Guides, etc.):
  - 2 pts for full disclosure how and where information is gathered for inclusion and disclosure on how that information was chosen for inclusion;
  - 1 pts for disclosure on how and where information is gathered for inclusion OR disclosure on how that information was chosen for inclusion;
  - 0 pts no disclosure on how and where information is gathered for inclusion nor disclosure on how that information was chosen for inclusion.
- Books:
  - 2 pts for provides complete list of names and credentials of contributing authors and disclosure of how authors and editors are recruited;
  - 1 pts for provides list of names and credentials of contributing authors and editors;
  - 0 pts for no information or very limited information for contributing authors and editors.
- Journals
  - 2 pts for provides complete list of editorial board members with contact information, clear author instructions which require disclosures for funding and conflict of interest, and an open data plan or open data statement;
  - 1 pts for provides complete list of editorial board members with contact information, clear author instructions for provides complete list of editorial board members with contact information, clear author instructions which require disclosures for funding and conflict of interest;
  - 0 pts for no information or very limited information for editorial board members and for author instructions.

## Q3. Are the contributors / creators of the resource experts in appropriate fields? *expert = degree and/or practice in the field that is being written in?

(1 pts = yes; 0 pts = no or not available)

## Q4. Is the content current?

(5 years = 2 pts; 10 years = 1 pts; 11+ years = 0 pts)

## Q5. ONLINE - Does the content get regularly updated?

(2 pts = if specified how often updated; 1 pts = with a date; 0 pts = undetermined)

## Q6. Funding disclosed?

(1 pts = yes; 0 pts = no or n/a)

### Scope Note for Q6.

- This applies to any discussion or disclosure of funding.
  - Books – If the authors and/or editors were given financial compensation for their contributions
  - Journals – If the item is OA, then how/when/if money is collected. If the journal is not OA, then it should be stated if the members of the editorial review board/reviewers are compensated or not. (A general statement is acceptable)
  - Online item – Funding is discussed for how/who provides funding
  - Database – If the database collects funding from governments/advertisers/etc., and/or is subscription, this should be stated clearly.

## Q7. Conflict of interest disclosed?

(1 pts = yes; 0 pts = no or n/a)

## Q8. Historically underrepresented groups are addressed / included?

(2 pts = addressed; 1 pts = included; 0 pts = no)

### Scope Note for Q8.

Historically Underrepresented Group refers to groups who have been denied access to resources (including health care), opportunities, and/or suffered past institutional discrimination in the United States and includes African Americans, Asian Americans, Hispanics or Chicanos/Latinos, and Native Americans and other ethnicities as well as Adult learners; Veterans; People with disabilities; LGBTQ+ individuals; Different religious groups, and different economic backgrounds. This resulted in marginalization for some groups and individuals and not for others, relative to the number of individuals who are members of the population involved. For more health care focused information please consider reading this page: <https://www.healthypeople.gov/2020/about/foundation-health-measures/Disparities>

- 2 pts if included and addressed: Historically Underrepresented Group(s) (HUG) are included in the resource AND the outcomes/issues/history/lack of or inadequate care are discussed, i.e. socioeconomic disparities in care, lack of information about LGBTQ+ health issues, historically based concerns with medical care for BIPOC communities, ect. Also, if HUG are acknowledged/discussed but not necessarily included in the item as HUG might be outside of the scope and/or no applicable information is available. I.e. resource on complimentary therapies in which data for people with disabilities is not available and the authors note that this is a gap in the literature that needs to be addressed;
- 1 pts if included: Resource includes information about/concerning Historically Underrepresented Group(s) (HUG), but does not provide in-depth acknowledgement of disparities in care. HUG are part of the resource, whether as part of the groups included in the resource or as the focus of the research, but historical disparities and/or current lack of research in area are not addressed;
- 0 pts if Historically Underrepresented Group(s) (HUG) not included/discussed at all.

## Q9. Is this intended for librarians or information professionals, or appropriate for a health sciences library collection?

(yes or no)
