# Supplementary material for: Using expert knowledge and peer review to create a reproducible process for the NAHRS Nursing Essential Resources List (NNERL)
Source: J Med Libr Assoc. 2025 Jan 14;113(1):78–84. doi: 10.5195/jmla.2025.1964 (PMC11835036; doi:10.5195/jmla.2025.1964)
Supplement: Supplementary file 2 — Appendix B: “Tags” with scope notes [file jmla-113-1-78-s02.docx]

**Table of Contents**

Format…………………………………………………………………………………………….2

Cost-Related to the Item…………………………………………………………………………………………..…..3

Areas of Interest/Topics…………………………………………………………………………………...4

Archival…………………………………………………………………………………………10

**Format**

Books

Non-periodical works are created to inform the audience. They can be read in both physical and electronic format and listened to in an audio format.

Databases

Collections of organized information are available electronically and structured in a way to facilitate the search and retrieval of information.

*Examples:*  PubMed/MEDLINE; ClinicalTrials.gov; CDC.gov; CINAHL; Alt HealthWatch; Cochrane Reviews

Journals

Periodic or serial publications containing articles written by experts in a defined field of study. They can be read in both physical and electronic format.

Serials

Publications are produced numerically or chronologically over several years and are generally intended indefinitely. They can be read in both print and electronic format.

Web Resources

Resources are available online via the Internet. They may or may not be available in a corresponding print or physical format.

**Cost-Related to the Item**

Subscription-Based

Items in which a cost is a recurring payment, or a recurring fee.

Free

Items in which there is no cost involved to view or interact.

One-Time Fee or Purchase

Items in which there is a one-time payment or need to be purchased to view or interact.

Freemium

Offering basic services or content for free while charging a fee for advanced or special features.

**Areas of Interest/Topics**

Archives

Collections of primary source materials that include, but are not limited to, historical documents, records, images, personal correspondence, and audio recordings. These items may be available in physical or electronic materials.

Bibliographies

Documents listing books, journals, and other materials on a subject or related subjects.

**Example:** NAHRS Essential Nursing Resources

Bioethics

The area of ethics focuses on biomedical and related areas of science, including nursing and health.

Complementary and Alternative Therapies

Therapies used in addition to or instead of conventional treatments which are used in Western medical approaches to care.

Consumer Health

Information about a health topic can be read, interpreted, or viewed and understood by the public, generally individuals without specialized training in nursing, medicine, or healthcare.

Continuing Education (CNE/CME)

Additional training for individuals to continue or advance their careers. The training can be either formal or informal. CNE is continuing nursing education. CME is continuing medical education.

Critical Appraisal

Systematically examining an item of information to determine the quality of the evidence presented.

Data Management

Collecting, maintaining, and using data securely and within current best practices.

Epidemiology and Statistics

The study and analysis of distribution and patterns for the determinants of health. It may include statistics on a disease.

Environmental Health

An area of healthcare focused on the role of the natural and built environment in promoting, establishing, and maintaining human health.

Evaluation and Assessment

The process to determine the quality and/or effectiveness of an organization, policy, plan, program, or method of attaining its specified goals or desired outcomes.

Evidence-Based Practice

A method or process of providing healthcare guided by integrating the best available scientific research evidence with clinical expertise and patient preferences.

Evidence Synthesis

A research method that combines information from multiple sources on a specific issue to identify all research on that topic and appraise and synthesize that research. The process should be conducted rigorously, unbiased, and reproducible.

Global Health

An area of healthcare focused on improving health and achieving equity in health for all people. It aims to transcend national boundaries when examining and addressing issues of both population-based and individual-based health needs.

Government (Created) Resources

Publications, including reports, infographics, and audiovisual material, are created by government agencies (local, state, national, or international).

Grant Resources

Non-repayable funding is awarded by an organization, government program, corporation, foundation, or trust to support research and other scholarly activities, initiatives, and programming.

Guidelines

A collection of directions or principles that provide guidance and/or policy for addressing a specific issue. Guidelines are generally created by an organization or a large group of professionals to unify treatments/approaches and are usually evidence-based.

**Examples:** US Preventative Task Force; Practice Guideline Resources (American Diabetes Association); Clinical Practice Guidelines

Health Disparities

Differences in accessibility, availability, and applicability in aspects of health experienced by people are often due to a lack of political, social, and/or economic power.

Health Literacy

A set of skills describes the ability to find, obtain, evaluate, and understand health information and then use the information gained to assist with making health decisions and guiding health behaviors.

Historically Underrepresented Groups (HUG)

Groups have been discriminated against, denied social and political power, and prevented from fully participating and utilizing resources due to their marginalized status.

History of Nursing

Information about the history and development of the field of nursing.

Informatics

Computer-based access, analysis, and dissemination of data.

Information Literacy

The ability to recognize when information is needed and have the skills to locate and evaluate material for accuracy and quality and the use of the information effectively.

Interprofessional

The practice (interprofessional practice) of having people from different disciplines, and the educational approach (interprofessional education) of having students from different majors, work together to solve a problem.

Measurements

Tools, scales, and other assessment instruments are used to obtain information about a population's characteristics and/or the outcomes of an intervention. A measurement can be validated, copyrighted, and/or open access.

**Examples:** Mental Measurements Yearbook; Tests in Print; HaPI [Health and Psychosocial Instruments]

Mental Health Resources

Focuses on an individual or group’s emotional, psychological, mental and social well-being.

Multidisciplinary

A resource that can be used by more than one discipline.

Nursing Administration

The management of nursing services involves processes such as planning, staffing, directing, budgeting, and supervising.

Nursing Models & Theories

Concepts, frameworks, methods, or assessments discuss definite behaviors or activities in nursing or nursing care. They can include various phenomena about nursing.

**Examples:** Nursing Theory (via <https://nursing-theory.org/>); Nursing Theory & Research (via <https://www.sandiego.edu/nursing/faculty-and-research/research/nursing-theory-research.php>)

Nursing Education

Entry-level and advanced practice educational pathways and materials are used by people who want to become nurses or who are practicing nurses.

Nursing Practice

Professional activities related to nursing and the performance of the duties associated with the provision of healthcare by nurses. These are based on scientific principles, established standards of care, and the nursing process.

Occupational Health

Area of healthcare focused on the safety, mental and physical health of employees in the workplace.

Patient Education

Healthcare professionals (e.g., nurses, doctors, and other healthcare practitioners) teach and educate patients to help them understand health information for themselves and apply it to their health situation. This can come in pamphlets, patient education sheets, consumer health websites, fact sheets, videos, consultations, or classes.

**Example:** MedlinePlus (<https://medlineplus.gov/>)

Patient Safety

The effort to reduce patient risks and prevent incidents and injuries in a healthcare setting. This can also include measures to reduce medical errors that are sometimes made by doctors, nurses, and other healthcare practitioners.

Pharmacology

The study of drugs and their interactions on living systems, more specifically, the study of how drugs work and interact in the body.

Point-of-Care

Tests and other healthcare services that are conducted at the bedside. Point-of-care systems or point-of-care tools refer to mobile applications used at the bedside or in a clinical setting to help diagnose, treat, or understand patients’ diseases and conditions.

*Examples:* UpToDate; DynaMed; Dynamic Health; Nursing Reference Center Plus

Professional Organizations

Groups that exist to advocate, support, and promote occupations where professionals pay a charge to be a member. Typically, the organization provides professional development opportunities, conferences, and other events to support the employees within the occupation’s ability to grow and develop their skills.

Public Health

An area of healthcare focused on the prevention and control of disease and the promotion of physical and mental health of a population on a municipal, state, national, or international level.

Quality Improvement

Framework used to improve healthcare systematically. This generally includes using standardized processes and structures and achieve predictable results to improve outcomes.

Quality of Healthcare

The degree to which healthcare services increase the likelihood of desired health outcomes is based on accepted quality standards and current professional knowledge.

Reference Materials

Provides background and general information on a particular subject. Designed to be consulted for specific information rather than to be read from cover to cover.

**Examples:** Dictionaries; Encyclopedias; Directories

Repositories

A place to host, store and maintain data.

Research Methods

The detailed process of research, including the procedure used to make or record systematic observations or collect data, evidence, or information that will be used to draw or make a conclusion based on the collected data, evidence, information, or observation.

Scholarly Communications

Materials and techniques that provide information about or are tools for scholarly and scientific data communication. This includes all processes connected to publication, open access, and information dissemination.

Scholarly Publishing

The act of publishing, presenting or creating scholarly content. Scholarly publishing is not paid to publish and is ideally performed to enhance the scientific conversation and preserve data, methods, and findings for future use.

Statistical Resources

Collections of large amounts of de-identified numerical data that are generally made available through a web-based portal, sometimes requiring permissions to access.

**Examples:** Robert Wood Johnson (<https://www.rwjf.org/>); Guttmacher Institute (<https://www.guttmacher.org>)

Toxicology

The study of toxic substances and poisons, including but not limited to their detection, composition, and biological actions and the treatment and prevention of toxic manifestation.

Writers Manuals and Guides

Writing manuals and guides describe how written materials should be arranged, cited, and written within a specific framework.

**Examples:** APA Style Guide; AMA Manual of Style

**Archival**

*Note:* Special “tag” to use if the item is not updated in 11 or more years but still part of the NAHRS Essential Titles list.

Archival

Resources are no longer updated or maintained but are included in the Essentials List for their historical importance. The resource has been taken down and cannot be accessed in some cases.
