# Supplementary material for: Using expert knowledge and peer review to create a reproducible process for the NAHRS Nursing Essential Resources List (NNERL)
Source: J Med Libr Assoc. 2025 Jan 14;113(1):78–84. doi: 10.5195/jmla.2025.1964 (PMC11835036; doi:10.5195/jmla.2025.1964)
Supplement: Supplementary file 3 — Appendix C: Additional Acknowledgements [file jmla-113-1-78-s03.docx]

# Appendix Three: Additional Acknowledgements

## Librarians Who Tagged or Scored Resources

Margaret Ansell, University of Florida​

Michelle Bass, Pennsylvania Hospital​

Marlowe Bogino, Rowan University/Cooper Medical Center​

Sunny Chung, Stony Brook University​

Jeannine Creazzo, Robert Wood Johnson University Hospital Somerset​

Bridget Gunn, Baystate Health​

Kimberly Harp, University of Nebraska Medical Center​

Elizabeth Huggins, Loyola University Chicago​

Sarah Katz, University of Delaware​

Sharon Leslie, Emory University​

Laura Lipke, Binghamton University

Elizabeth Morgan, UPMC​

Ryan Norman, Robert Wood Johnson University Hospital Somerset​

Lisa Lian Philpotts, Massachusetts General Hospital​

Jamie Quinn, Baylor University - Louise Herrington School of Nursing, Dallas​

Aida Marissa Smith, Ascension, Nursing Center of Excellence​

Marisa Testerman, The University of Texas at El Paso​

Faythe Thurman, West Virginia University​

Jennie Ver Steeg, Mercy College of Health Sciences​

## Librarians Who Were Original Members of Task Force

Tara Brigham, Mayo Clinic

Anne Foster, Mclaren Health Care

Mikaela Gray, University of Toronto

Amanda Haberstroh, East Carolina University

Halyna Liszczynskyj, Mohawk Valley Health System

Patrice O'Donovan, Linfield University

Kat Phillips, Penn State University

Mary Rickelman, Advent Health University​ (also tagged resources)

Susan Robins, SUNY Downstate Health Sciences University

Jessica Sender, Michigan State University

Kitty Serling, Institute for International Medicine

Claire Sharifi, University of San Francisco

Rachel L. Walden, Vanderbilt University
